# Chemotherapy Completion as a Quality Metric in Resected Pancreatic Ductal Adenocarcinoma

**DOI:** 10.3390/cancers18121912

**Published:** 2026-06-11

**Authors:** Robert C. G. Martin, Ryan A. Cantrell, Jeremy T. Gaskins

**Affiliations:** 1 The Hiram C. Polk, Jr., MD Department of Surgery, Division of Surgical Oncology, University of Louisville School of Medicine, 315 E. Broadway, Louisville, KY 40292, USA; ryan.cantrell@louisville.edu; 2 Department of Bioinformatics & Biostatistics, University of Louisville School of Medicine, Louisville, KY 40292, USA; jeremy.gaskin@louisville.edu

**Keywords:** pancreatic adenocarcinoma, optimal therapy, overall survival, chemotherapy, outcomes

## Abstract

**Background**: Over 60,000 patients are diagnosed annually with pancreatic cancer in the United States, most with pancreatic ductal adenocarcinoma (PDAC). Despite multimodality treatment, prognosis remains poor, underscoring the need to better define factors associated with improved survival in resectable disease. The aim of this study was to propose “completeness of therapy” in resectable PDAC based on chemotherapy type, relative dose intensity (RDI), duration, and timing of initiation, and to evaluate their association with overall survival (OS). **Methods**: A systematic review of PubMed identified 34 studies. Hazard ratios (HRs) were extracted and synthesized in forest plots. Outcomes focused on OS in relation to chemotherapy completion (cumulative cycles), time to adjuvant chemotherapy initiation (TTA), RDI, and regimen type. The goal was to conceptualize an evidence-based completeness-of-therapy framework for resected PDAC. **Results**: Completion of chemotherapy was associated with a 42% reduction in the hazard of death compared with incomplete therapy (HR = 0.58, 95% CI 0.47–0.71, *p* < 0.01). No significant OS difference was observed for longer TTA within a ≤12-week window after surgery (HR = 1.22, 95% CI 0.95–1.56, *p* = 0.10). Higher RDI demonstrated a large but non-significant trend toward improved OS (HR = 0.51, *p* = 0.24). Non-significant trends favoring gemcitabine-based regimens (HR = 0.87, *p* = 0.26) and FOLFIRINOX (HR = 0.79, *p* = 0.26) were observed, with FOLFIRINOX suggesting a 21% relative reduction in mortality. **Conclusions**: Chemotherapy completion is strongly associated with improved OS in resectable PDAC. Initiation of adjuvant therapy within 12 weeks appears sufficient, allowing recovery from surgery. Higher RDI and specific chemotherapy regimens demonstrated numerically favorable hazard ratios; however, these associations were not statistically significant and should be interpreted cautiously.

## Simple Summary

Pancreatic ductal adenocarcinoma (PDAC) is a highly lethal cancer, with most patients ultimately dying from the disease. This study reviewed published research to understand how the completeness of adjuvant chemotherapy after surgical removal of PDAC affects survival. Across 34 studies, patients who completed their planned chemotherapy had a 42% lower risk of death compared with those who did not. Starting chemotherapy within 12 weeks after surgery did not significantly affect survival, suggesting treatment should begin once patients have adequately recovered. Higher chemotherapy dose intensity and specific regimens (gemcitabine-based or FOLFIRINOX) showed trends toward improved survival, though these findings were not statistically significant. Overall, the results emphasize that completing chemotherapy is the most important factor associated with improved survival in patients with resected PDAC.

## 1. Introduction

One of the advances in the treatment of pancreatic adenocarcinoma has been the benefit of adjuvant and neo-adjuvant (NAC) chemotherapy in reducing recurrence and improving overall survival [1,2]. Approximately 60,000 patients are estimated to receive a new diagnosis of pancreatic cancer in the USA with pancreatic ductal adenocarcinoma (PDAC) being the dominant pathology [1]. Unfortunately, all are expected to eventually succumb to this diagnosis in the following months/years, even those who receive a microscopically negative resection. The poor prognosis associated with resected PDAC has been attributed to the high rates of distant and local recurrence (80% and 20%, respectively). Improvements in overall outcomes have occurred with several trials in the past decades investigating the role of multimodality approaches to improve the disease control rates in PDAC. The survival benefit of adjuvant chemotherapy (ACT) in resected PDAC was evident in the early CONKO-001 [3] and ESPAC-3 [4] trials establishing the key role of gemcitabine-based ACT. Later trials even suggested superiority of other regimens such as FOLFIRINOX in the PRODIGE-24 trial [5]. However, the additional benefit of sequential chemoradiotherapy (CRT) or adjuvant radiotherapy (ART) alone remained unclear in resected PDAC; hence, the current recommended treatment algorithms include ACT alone, ACT followed by CRT, or CRT in between cycles of ACT [6]. Multiple studies have attempted to evaluate the benefit of CRT in the adjuvant setting but unfortunately, all experienced major flaws in their study design, target patient population, and analysis, which limited their interpretation and contributed to a lack of consensus [7,8].

The optimal sequencing of multimodality therapy for resectable pancreatic ductal adenocarcinoma (PDAC) remains an area of active investigation. While several contemporary studies have suggested potential benefits of neoadjuvant chemotherapy, even among anatomically resectable tumors, others have emphasized the continued importance of surgical quality, margin status, and adequacy of mesopancreatic dissection in determining oncologic outcomes. Recurrence following resection likely reflects a complex interaction between tumor biology, occult micrometastatic disease, treatment delivery, and surgical factors including achievement of a truly radical resection. Although these issues remain highly relevant to outcome optimization, the present review focuses specifically on chemotherapy delivery metrics—including treatment completion, timing, relative dose intensity, and regimen selection—and their association with survival following curative-intent resection.

With the inclusion of multiple treatment options in the management of PDAC, there has not been an “optimal” treatment(s) proposed in resectable PDAC. The aim of this study was to evaluate and propose the overall optimal therapy in Stage 2b or earlier pancreatic adenocarcinoma. Surgical quality standards have been well defined for textbook outcomes [9], estimated blood loss [10], surgical margins [11], lymphadenectomy [12], 30- and 90-day mortality [13], and readmission rates [10,14] and thus are well established and transparent quality metrics. Despite increasing use of textbook surgical outcomes, no analogous framework exists for systemic therapy delivery. The understanding of the completeness of therapy based on chemotherapy type, duration of chemotherapy, chemotherapy dose intensity, initiation of therapy, and other factors have not been proposed in relation to disease-free survival and overall survival [15,16]. The aim of this study is to bridge surgical success with oncologic delivery through a review of the current literature for optimal chemotherapy in pancreatic adenocarcinoma.

## 2. Methods

### 2.1. Literature Search

A systematic review was conducted in accordance with the Preferred Reporting Items for Systematic Reviews and Meta-Analyses (PRISMA 2020) guidelines. Electronic database searches were performed in PubMed/MEDLINE, Embase (OvidSP), Web of Science Core Collection, Library of Congress, and LISTA (EBSCO) from 1 January 2015 through 31 December 2023. This review was a registered systematic review in PROSPERO (CRD420261375555).

The search strategy combined controlled vocabulary and free-text terms related to pancreatic ductal adenocarcinoma and perioperative systemic therapy. Core search terms included:

“pancreatic adenocarcinoma” OR “pancreatic ductal adenocarcinoma” OR “PDAC”

“adjuvant chemotherapy”

“neoadjuvant chemotherapy”

“chemotherapy completion”

“relative dose intensity”

“time to adjuvant chemotherapy”

“FOLFIRINOX”

“gemcitabine”

“pancreaticoduodenectomy”

Boolean operators (AND/OR) and database-specific indexing terms were applied when appropriate. Reference lists of eligible articles and relevant review manuscripts were manually screened to identify additional studies.

Search results were imported into a reference management system and duplicate records were removed prior to screening.

**Figure 1:**
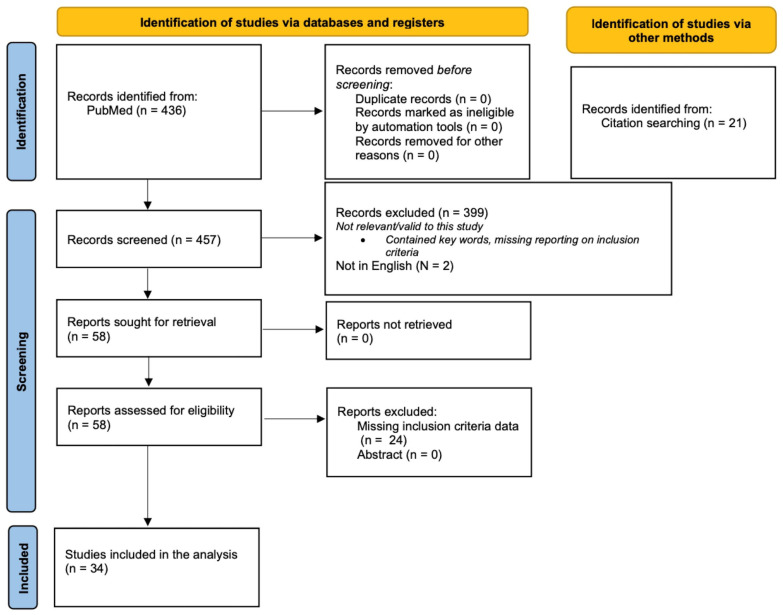
Preferred Reporting Items for Systematic Reviews and Meta-Analyses (PRISMA) diagram of literature search. PICO, patient/population, intervention, comparison and outcomes.

Studies were eligible if they:

Included patients with resected pancreatic ductal adenocarcinoma;

Reported perioperative systemic chemotherapy variables;

Evaluated outcomes related to overall survival (OS) and/or disease-free survival (DFS);

Reported at least one treatment-delivery variable including chemotherapy completion, cumulative cycles, time to adjuvant chemotherapy initiation (TTA), relative dose intensity (RDI), or chemotherapy regimen;

Were published in English. Studies lacking survival outcomes or insufficient treatment-delivery data were excluded (Figure 1).

### 2.2. Study Selection

The study selection process followed PRISMA methodology. Following database searching and manual reference review, 457 records were identified. After duplicate removal and title/abstract screening, 399 studies were excluded for failure to meet predefined eligibility criteria. Two additional studies were excluded due to non-English publication.

Full-text review was performed for 58 potentially eligible articles. Of these, 24 studies were excluded because they lacked survival outcomes or did not report relevant treatment-delivery variables including chemotherapy completion, TTA, RDI, or regimen-specific data. Ultimately, 34 studies met inclusion criteria and were included in qualitative synthesis, with subsets eligible for quantitative meta-analysis depending on hazard ratio availability and outcome definition (Figure 1). Quality metrics for surgical resection were excluded for this review since these established metrics of operative time, estimated blood loss, pancreatic leak, bile leak, delayed gastric emptying, 30- and 90-day mortality, and re-admission have all been established and agreed upon in previous manuscripts.

### 2.3. Data Extraction and Quality Assessment

Two authors independently screened the titles and abstracts of all articles identified in the primary and secondary search strategy. Based on the inclusion and exclusion criteria, the authors assessed the full text of 34 articles, and then subsequently performed the data extraction (R.C. and RCGM). The data extracted was focused on outcomes related to improved disease-free survival (DFS) and overall survival (OS) relating to the following variables: cumulative number of chemotherapy cycles, number of postoperative weeks until initiation of adjuvant chemotherapy, relative dosage intensity percentage, and chemotherapy regimen type. These four variables were chosen because they were the most frequently reported on chemotherapy treatment variables in the literature to have an impact on patient DFS or OS. The primary endpoint was to suggest an optimal completeness of therapy model to support a framework of the minimal medical treatment a patient should receive for their optimal completeness of therapy after resection of their pancreatic adenocarcinoma.

### 2.4. Study Scoring

A scoring system adapted from the methodological index for non-randomized studies (MINORS) [17] was implemented to generate a study score. For non-randomized studies points are awarded for eight different categories. Zero points for a category not being reported, one point for reported but inadequate, and two points for reported and adequate. The eight categories include a clearly stated aim, inclusion of consecutive patients, prospective collection of data, endpoints appropriate to the aim of the study, unbiased assessment of the study endpoint, follow-up period appropriate to the aim of the study, loss to follow up less than 5%, and prospective calculation of the study size. An additional four categories are added for a comparative study. These include an adequate control group, contemporary groups, baseline equivalence of groups, and adequate statistical analysis.

Risk of bias was assessed independently by two reviewers using the Methodological Index for Non-Randomized Studies (MINORS) instrument for observational studies. Each study was evaluated across eight domains for non-comparative studies and twelve domains for comparative studies, with individual domains scored as

- 0 = not reported;
- 1 = reported but inadequate;
- 2 = reported and adequate.

Domains evaluated included clearly stated study aim, consecutive patient inclusion, prospective data collection, endpoint appropriateness, unbiased endpoint assessment, adequate follow-up, loss to follow-up, prospective sample size calculation, and comparative design elements when applicable. Studies were categorized as low, moderate, or high methodological quality according to total MINORS score. Quality assessment results are summarized in Supplementary Table S1 and were considered during interpretation of pooled findings.

Disagreements between reviewers were resolved by consensus. MINORS scores were summarized descriptively and used to characterize methodological quality but were not used as exclusion criteria or weighting factors within meta-analysis.

### 2.5. Statistical Analysis

Studies that included comparisons in overall survival based on the treatment characteristics were eligible for inclusion in meta-analysis. Manuscripts must have used a binary/categorical variable in the Cox model for OS, as hazard ratios (HR) based on continuous version of the treatment characters cannot be easily compared. Univariate HRs were used when available, and adjusted HRs from multivariate models were used if that was the only HR reported. To ensure HRs were considered relative to the same reference group, HRs were inverted if reported using the opposite reference groups; for studies that reported effects relative to a reference group not of interest to our analysis (i.e., no treatment), HRs were estimated by obtaining the relevant contrast of the log(HR) and using a conservative estimate of the variance from summing the variance of each log(HR) estimate. Meta-analysis was performed by combining the log (HR)s of all studies meeting the above criteria using the standard random-effects model to account for heterogeneity across studies. Forest plots included the estimated combined HR, confidence interval, *p*-value from the overall test and the I^2^ heterogeneity statistics are reported. This statistical analysis was performed using R statistical software, version 4.2.2.

### 2.6. Publication Bias Assessment

Publication bias was evaluated when sufficient studies (≥10) were available for pooled analysis. Funnel plots were visually inspected for asymmetry, and Egger’s regression test was used to statistically evaluate small-study effects. Due to the limited number of eligible studies in several analyses, formal publication bias assessment was not feasible for all outcomes and findings should therefore be interpreted cautiously.

## 3. Definition of Completeness of Therapy Framework

Because no standardized definition of “completeness of therapy” exists for resected pancreatic ductal adenocarcinoma, a hierarchical conceptual framework was developed based on the strength and consistency of available evidence.

Treatment-delivery variables were organized into three tiers:

**Tier 1 (Core determinant):**

- Completion of systemic chemotherapy, defined most commonly as receipt of ≥6 cumulative cycles and supported by the strongest survival evidence.

**Tier 2 (Delivery modifiers):**

- Time to adjuvant chemotherapy initiation (TTA);
- Relative dose intensity (RDI).

These variables may influence treatment completion and survival but demonstrated less consistent associations across studies.

**Tier 3 (Clinical context variables):**

- Chemotherapy regimen selection (e.g., gemcitabine-based or FOLFIRINOX-based therapy).

Regimen choice was considered contextual rather than determinative, reflecting feasibility, patient selection, and treatment tolerance.

This hierarchical framework was used to organize synthesis and conceptualize completeness of therapy following PDAC resection.

Because no validated definition currently exists, the proposed framework should be considered conceptual and hypothesis-generating. Variable definitions were extracted as reported by individual studies, and the framework was developed to organize existing evidence rather than establish a universal clinical standard.

## 4. Results

### Duration/Completeness of Chemotherapy: Neo-Adjuvant or Adjuvant

Of the 34 articles selected, 23 reported on the number of cycles received before or after pancreatectomy. The reporting on this data varied, and 22 of the articles reported their definition of completeness as a hard cutoff (i.e., >4 cycles, >6 cycles or >10 cycles) or the least number of cumulative cycles (i.e., <4 cycles of <6 cycles) that must be reached to be considered a complete therapy [18,19,20,21,22,23,24,25,26,27,28,29,30,31,32,33,34,35,36,37,38,39,40,41]. The remaining article reported their data as median number of cycles received [42].

Our systematic review found 12 articles reporting the number of cycles about OS, eight articles regarding disease free survival (DFS) and the last three with just the number of cycles found in their analysis (Table 1). The most frequently reported number of cycles of optimal effective chemotherapy was a N = 6; thus, the findings can most easily be reported as studies where patients received less than six cycles or at least six cycles. Studies that report receiving less than six cycles of chemotherapy were found in two of the articles [19,25]. Studies that reported on receiving at least six cycles of chemotherapy, were found in 21 articles [4,18,20,21,22,23,24,26,27,28,29,30,31,32,34,35,36,39,40,41,42]. Of the 21 articles receiving at least six cycles, 12 reported an increased survival advantage associated with the receipt of at least six cumulative chemotherapy cycles compared to less than six cycles. A total of seven out of 12 articles found a significant survival advantage with the receipt of at least six chemotherapy cycles [21,22,23,24,30,34,40], and the remaining five articles reported a non-significant improved OS associated with six cycles [18,28,29,32,41]. Thus, based on this data receiving at least six cycles of cumulative chemotherapy has a survival advantage for patients with resectable pancreatic adenocarcinoma.

**Figure 2:**
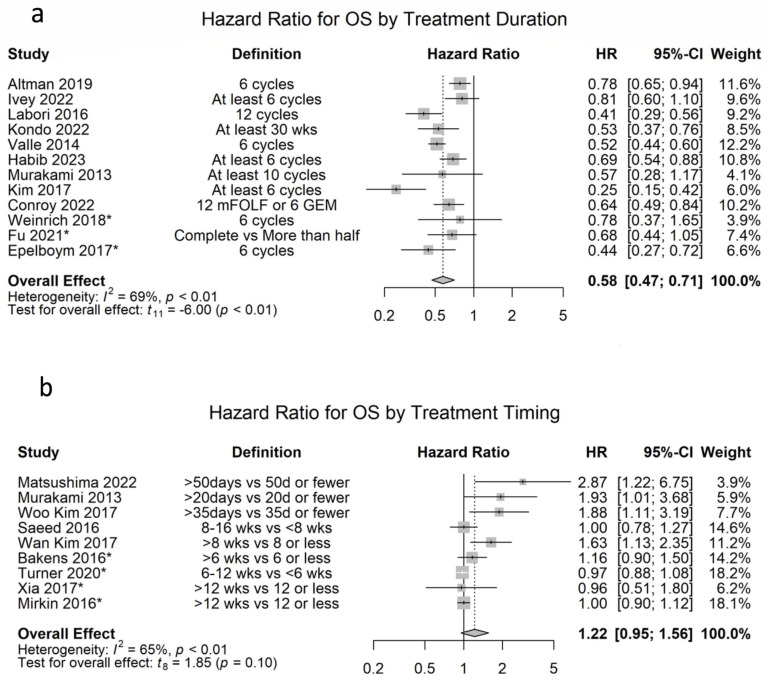
(**a**) Forest plot of manuscripts reporting the overall survival in pancreatic adenocarcinoma based on the duration or number of cycles of chemotherapy in the neo-adjuvant and/or adjuvant time interval. The “*” in the paper names indicate that the HR has been estimated using the HRs from the papers, e.g., Weinrich compares complete and incomplete to no AC, and we compute the HR for complete vs. incomplete. (**b**): Forest plot of manuscripts reporting the overall survival in pancreatic adenocarcinoma based on the time to initiating or re-initiating adjuvant therapy.

Meta-analysis was considered for the 12 manuscripts that reported a hazard ratio for OS by treatment competition (as defined within each manuscript). Despite moderately high heterogeneity (I^2^ = 69%), the combined results indicate that patients completing CT (>6 cycles of chemotherapy) have a 32% lower risk of death (HR = 0.58, 95% CI 0.47–0.71, *p* < 0.01; Figure 2). This association between number of cycles and OS was individually significant within eight of the 12 manuscripts [18,20,22,23,24,30,34,40].

## 5. Time to Adjuvant Chemotherapy

Our systematic review of 34 articles found that 24 reported data on the time to adjuvant (TTA) chemotherapy post pancreatectomy. The data was reported in multiple ways including the median TTA (median 7 weeks) or greater/less than multiple time intervals (Table 2). Due to the variable nature of the reporting and subsequent results the timing data can most easily be report on as TTA ≤ 12 weeks or >12 weeks. All 24 articles reported an effect of TTA that fell within this ≤12 weeks evaluation criteria [4,15,16,18,20,21,22,23,25,32,33,34,35,36,37,39,41,43,44,45,46,47,48,49]. If the data was reported with a reference to impact on survival that data was pulled and assembled into Table 2. A total of 12 articles reported on TTA of ≤12 weeks with reference to OS within this period. Of these 12 articles, a total of two found a significant hazard ratio associated with improved overall survival starting TTA within the ≤12-week window [43,46]. Of these 12 articles, a total of five found a significant hazard ratio associated with worsened overall survival starting TTA within the ≤12-week window [15,25,32,33,34]. The remaining six articles reported non-significant overall survival hazard ratios for TTA ≤ 12 weeks [15,16,23,35,36,37]. The remaining 12 articles with a TTA ≤ 12 weeks reported their data with no reference to the impact of TTA and survival. The 12-week threshold was selected because it represented the most commonly reported clinically relevant cutoff across included studies and aligns with timing windows evaluated in major adjuvant chemotherapy trials and population-based analyses. However, definitions varied across studies, contributing to heterogeneity and limiting interpretation of any single threshold.

## 6. Time to Adjuvant Meta-Analysis

**Figure 3:**
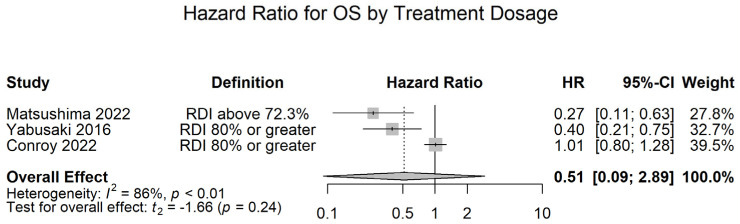
Forest plot of manuscripts reporting the overall survival in pancreatic adenocarcinoma based on the relative dose intensity (RDI) of chemotherapy.

OS HR data pertaining to the TTA was extracted from the screened articles, and nine articles contained data regarding a hazard ratio for OS based on a binary definition of TTA. Meta-analysis on this data failed to show a significant association between treatment timing and OS (HR = 1.22, 95% CI 0.95–1.56, *p* = 0.10) (Figure 3), although trended towards poorer survival for patients with delayed treatment initiation. Heterogeneity in this setting was moderately high (65%) with one key driver potentially being differences in definition of delayed treatment timing across studies. Four of the nine articles showed an increased HR associated with a longer TTA [25,33,34].

## 7. Relative Dosage Intensity

Of the four identified variables in Table 2, RDI was reported far less frequently. In the literature review three articles were found that studied the effect of RDI on OS. Data on RDIs was pulled and assembled into Table 2. Matsushima et al. found >72.3% RDI was significantly associated with improved OS [25], and similarly Yabusaki et al. found a RDI of ≥80% was significantly associated with improved OS [26]. Conroy et al. found no significant association between ≥80% RDI and OS [40].

**Figure 4:**
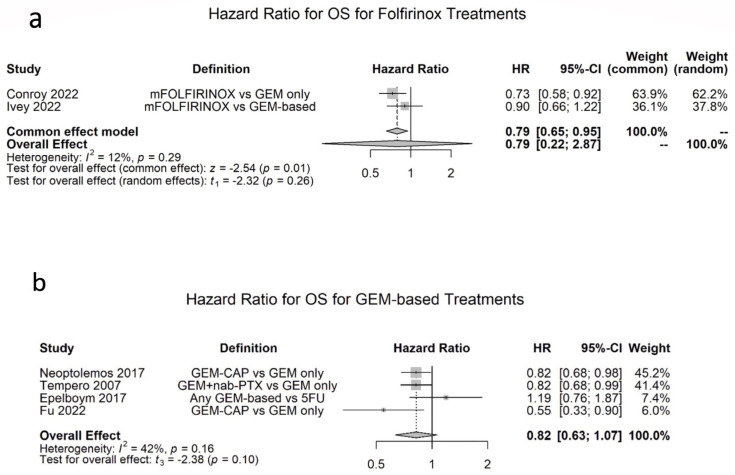
(**a**) Forest plot of manuscripts reporting the overall survival in pancreatic adenocarcinoma based on the FOLFIRINOX chemotherapy. (**b**) Forest plot of manuscripts reporting the overall survival in pancreatic adenocarcinoma based on the GEM-based chemotherapy.

Meta-analysis with these three studies to compare the relationship between high-intensity dose and OS was performed (Figure 4). Heterogeneity in case was very high (86%). The combined results suggest patients with high treatment dose may be at substantially reduced risk of death, but with only three studies and high heterogeneity, this effect is not statistically significant (HR = 0.51, 95% CI 0.09–2.89, *p* = 0.24).

## 8. Chemotherapy Regimen

Of the 34 articles 28 reported data on the chemotherapy regimen used. Any data reporting on chemotherapy regimen was extracted and assembled into Table 2. It was frequently reported that either a FOLFIRINOX regimen or Gemcitabine (GEM)-based regimen was used. Thus, the articles were split into two groups, the first being the inclusion of FOLFIRINOX or GEM-based regimen, and the second group includes any other regimen reported. A total of 25 articles reported using either FOLFIRINOX or a GEM-based regimens. The three remaining articles did not incorporate FOLFIRINOX or GEM-based regimens; they reported using S-1, FU+LV, fluoropyrimidine-based, or oxaliplatin- based. Of articles reporting the use of FOLFIRINOX or GEM-based regimens nine reported the impact of these regimens on survival. A total of five articles reported a significantly increased survival advantage for the use of either FOLFIRINOX or GEM-based regimens [27,38,39,40,43]. The remaining four articles found no significant difference on OS between a FOLFIRINOX or GEM-based regimen [19,24,26,30]. The remaining 19 FOLFIRINOX or GEM-based articles had no reference to the impact of regimen type on survival.

### 8.1. GEM-Based Regimen Meta-Analysis

A total of five articles included OS HR for a GEM-based regimen compared to any chemotherapy regimen historically used for PDAC. GEM-based is defined as GEM+ a commonly used regimen for PDAC. GEM-based regimens did not display a survival advantage in the meta-analysis (HR = 0.87, 95% CI 0.65–1.17, *p* = 0.26). Note that in the three manuscripts comparing GEM-based to GEM only, the GEM-based regimen was found to show a statistically significant reduction in hazard of death. When compared to 5FU and mFOLFIRINOX, GEM-based had poorer, but not statistically significant, survival.

### 8.2. FOLFIRINOX Regimen Meta-Analysis

Only two articles could be found reporting OS HR pertaining to FOLFIRINOX vs. any chemotherapy regimen historically used for PDAC. The usual random-effects meta-analysis suggests a survival improvement of 21% (HR = 0.79, 95% CI 0.22–2.87, *p* = 0.26), but due to the small sample size, this is not statistically significant. Considering the small sample and low estimated heterogeneity (I^2^ = 12%), we also consider a fixed-effects model. Although the fixed-effects model suggested a potential survival benefit for mFOLFIRINOX, only two studies were available for analysis. Given the limited sample size, observational design, and lack of significance in the random-effects model, these findings should be evaluated with these limitations.

## 9. Discussion

Despite advances in surgical technique and systemic therapy, long-term outcomes for patients with resected pancreatic ductal adenocarcinoma (PDAC) remain poor. While surgical quality metrics are now well defined and widely accepted, there is no analogous framework describing the optimal delivery or completeness of perioperative chemotherapy. This systematic review and meta-analysis sought to synthesize the available literature examining chemotherapy duration, timing of initiation, relative dose intensity, and regimen type in order to better characterize treatment delivery factors associated with overall survival (OS) following resection. Currently there is no completeness of treatment standard of care conceptualized for PDAC, which leaves patients treatment largely up to the variable discretion of the physician [31].

The most consistent and robust finding of this analysis is the association between completion of planned chemotherapy, most commonly defined as receipt of at least six cumulative cycles within the literature [18,20,21,22,23,24,26,27,28,29,30,31,41,42,50,51], with its association to improved RFS and OS. Across multiple retrospective cohorts and supported by meta-analysis, patients who completed chemotherapy experienced a substantially lower hazard of death compared with those who did not. This finding was observed regardless of whether chemotherapy was delivered in the neoadjuvant or adjuvant setting, suggesting that cumulative systemic exposure—rather than sequencing alone—may be a critical determinant of long-term outcomes. Importantly, chemotherapy completion likely reflects a combination of favorable tumor biology, adequate postoperative recovery, and preserved patient performance status, rather than a purely treatment-driven survival effect. Nevertheless, completion of systemic therapy emerges as a reproducible and clinically meaningful benchmark that warrants consideration as a quality metric in resected PDAC care.

In contrast, the relationship between time to initiation of adjuvant chemotherapy (TTA) and survival was less consistent. Although delayed initiation of adjuvant therapy has been associated with worse outcomes in several other malignancies [16,52,53,54,55], the data in PDAC remain mixed [23,32,37]. In this meta-analysis, initiation of chemotherapy within a 12-week window following resection was not significantly associated with OS, despite moderate heterogeneity across studies. These findings support prior observations from randomized trials and population-based analyses suggesting that early initiation of chemotherapy may not confer a survival advantage if patients have not adequately recovered from surgery. Collectively, these data reinforce a patient-centered approach to TTA, emphasizing physiological recovery and treatment tolerance rather than rigid adherence to an early postoperative timeline.

Relative dose intensity (RDI) represents a potentially important but underexplored aspect of chemotherapy delivery in PDAC. Only three studies met inclusion criteria for analysis, with heterogeneous definitions of “high” RDI and substantial between-study variability. Although individual studies suggested improved survival with higher dose intensity, the pooled estimate was not statistically significant [25,26]. Given the limited number of studies and high heterogeneity, the current evidence is insufficient to define RDI thresholds as part of a standard completeness-of-therapy model. Nonetheless, these findings highlight an important gap in the literature and suggest that maintaining adequate dose intensity may warrant further investigation in prospective studies. The importance of RDI is related to cancer recurrence after curative surgery likely arising from residual micro metastases; thus, the administration of sufficient ACT following surgery may be required to eradicate residual cancer cells [25]. Both Yabusaki et al. and Matsushima et al. found RDIs >70% are associated with improved OS. Although point estimates favored higher RDI, the pooled analysis did not demonstrate statistical significance and was limited by substantial heterogeneity and small sample size. With the lack of other evidence in the literature any conclusion based on these two studies is not definitive. An effort to conduct further studies on RDI and is association with OS in PDAC patients must be conducted to further this hypothesis of RDI being included in an optimal completeness of therapy model.

Evaluation of chemotherapy regimen type similarly yielded limited conclusions. While randomized trials have clearly demonstrated superiority of modern regimens such as gemcitabine–capecitabine and modified FOLFIRINOX compared with older standards, regimen-specific survival comparisons in real-world observational studies were sparse and underpowered. More recently RCTs have tested the efficacy of GEM-based and FOLFIRINOX regimens for PDAC applications. The ESPAC-4 trial [38] established that GEM+capecitabine (GEM-CAP) ACT has superior OS than GEM monotherapy. Around the same time the PRODIGE-24 trial [5] compared mFOLFIRINOX vs. GEM monotherapy yielding superior DFS for the mFOLFIRINOX arm. Meta-analyses of gemcitabine-based and FOLFIRINOX regimens did not demonstrate statistically significant survival advantages, largely due to small sample sizes, heterogeneity in comparator regimens, and confounding by patient selection. These findings should not be interpreted as contradicting randomized trial evidence, but rather underscore the challenges of evaluating regimen efficacy in retrospective cohorts.

Taken together, this study suggests that successful delivery and completion of systemic therapy is the most reliable chemotherapy-related factor associated with improved survival in resected PDAC. Other treatment characteristics—including timing, dose intensity, and regimen choice—appear to play contributory roles but are less consistently supported by the current literature. These findings support a paradigm shift toward viewing chemotherapy completion as a pragmatic, patient-centered quality benchmark, rather than focusing on individual treatment components in isolation.

**Figure 5:**
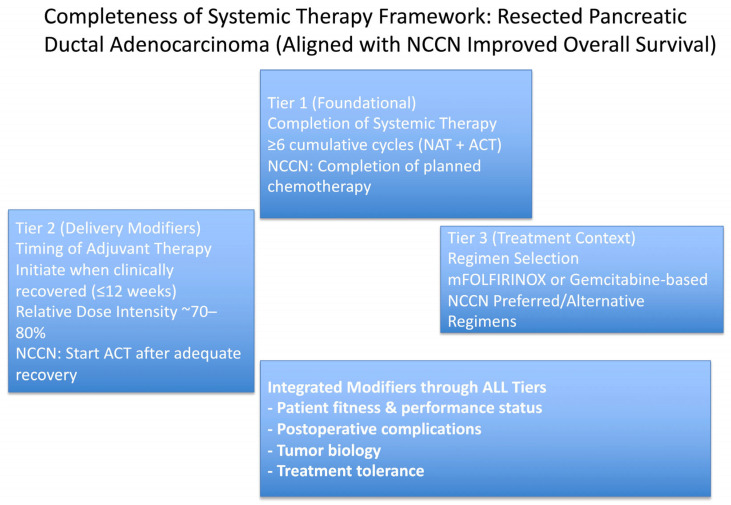
Conceptual framework defining completeness of systemic therapy following resection of pancreatic ductal adenocarcinoma. Completion of chemotherapy (≥6 cumulative cycles delivered in the neoadjuvant and/or adjuvant setting) represents the most robust and reproducible treatment-related factor associated with improved overall survival. Timing of therapy initiation and relative dose intensity function as delivery modifiers that influence the ability to complete therapy but demonstrates less consistent independent associations with survival. Chemotherapy regimen selection provides the clinical context for treatment delivery but appears to influence outcomes primarily through feasibility of completion rather than regimen choice alone. Patient fitness, postoperative recovery, and tumor biology act as cross-cutting modifiers across all domains.

Based on these findings, we propose a multidimensional framework for “completeness of therapy” in resected PDAC, in which completion of systemic chemotherapy represents the foundational determinant most strongly associated with survival (Figure 5). Timing of initiation and relative dose intensity function as treatment delivery modifiers that influence the likelihood of completion, while chemotherapy regimen selection provides necessary clinical context. Importantly, patient fitness, postoperative recovery, and tumor biology intersect across all domains, underscoring that completeness of therapy should be viewed as a pragmatic quality benchmark rather than a prescriptive treatment mandate.

This study has several important limitations that must be considered when interpreting the results. First, the majority of included studies were retrospective and observational, introducing inherent risks of selection bias, confounding by indication, and immortal time bias. Patients who complete chemotherapy are, by definition, those who survive long enough and remain fit enough to do so; therefore, chemotherapy completion may serve as a surrogate marker for favorable tumor biology, postoperative recovery, and baseline performance status rather than a direct causal determinant of survival. Second, there was substantial heterogeneity in study populations, treatment paradigms, and variable definitions. Studies variably included surgery-first, neoadjuvant, and mixed perioperative approaches, limiting the ability to draw sequencing-specific conclusions. Definitions of chemotherapy completion, delayed treatment initiation, and high relative dose intensity were inconsistent across studies, contributing to heterogeneity in pooled analyses. Third, several meta-analyses were limited by small numbers of eligible studies, particularly those evaluating relative dose intensity and chemotherapy regimen type. As a result, these analyses were underpowered and should be considered hypothesis-generating rather than definitive. Additionally, adjusted hazard ratios were not uniformly available, and univariate estimates were used in some cases, further limiting causal inference. Finally, patient-level data were not available, precluding adjustment for important confounders such as postoperative complications, comorbidity burden, performance status, and pathological risk factors. These limitations are intrinsic to the existing literature and highlight the need for prospective studies specifically designed to evaluate chemotherapy delivery metrics in resected PDAC.

Additional limitations warrant consideration. First, substantial inter-study heterogeneity existed across included cohorts with respect to chemotherapy regimens, treatment sequencing, staging systems, patient selection criteria, and definitions of treatment-delivery variables. Although random-effects meta-analysis was used to account for heterogeneity, residual confounding likely remains. Second, pooled hazard ratios were derived from published estimates, which varied in the degree of covariate adjustment across studies. Consequently, observed associations may reflect differences in analytical approaches rather than treatment effects alone. Third, potential overlap of patient populations among large registry-based and multi-institutional studies cannot be completely excluded, although duplicate publications were screened during study selection. Finally, the absence of patient-level data prevented standardized adjustment for important prognostic factors including performance status, postoperative complications, margin status, nodal burden, and receipt of neoadjuvant therapy. Grey literature, conference abstracts, and unpublished studies were not included, which may increase susceptibility to publication bias.

An additional limitation is that surgical variables were not uniformly reported across included studies. Important factors such as operative technique, extent of lymphadenectomy, mesopancreatic excision, margin assessment methodology, posterior margin status, and postoperative complications varied substantially or were unavailable in many reports. Consequently, the influence of surgical quality and operative radicality on chemotherapy completion and survival outcomes could not be independently assessed. The present analysis therefore should be interpreted as an evaluation of chemotherapy delivery metrics rather than a comparative assessment of surgical strategies or treatment sequencing approaches.

## 10. Conclusions

In this systematic review and meta-analysis, completion of planned chemotherapy—most commonly defined as receipt of at least six cumulative cycles—was the strongest and most consistent treatment-related factor associated with improved overall survival in patients with resected pancreatic ductal adenocarcinoma. In contrast, time to initiation of adjuvant chemotherapy within 12 weeks of surgery was not independently associated with survival, supporting individualized, recovery-based treatment initiation. Evidence regarding relative dose intensity and chemotherapy regimen remains limited and insufficient to define firm treatment thresholds. These findings suggest that future quality frameworks in pancreatic cancer care should extend beyond surgical metrics to incorporate successful delivery and completion of systemic therapy as a key benchmark. Prospective, patient-level studies are needed to validate chemotherapy completion as a modifiable quality indicator and to better define how timing, dose intensity, and regimen selection interact to optimize outcomes in resected PDAC. Chemotherapy completion is the next evolution beyond textbook outcomes.

## Figures and Tables

**Table 1:** Selected articles presented based on MINORS study scoring quality and cumulative chemotherapy cycles.

Author	Publication Year	MINORS Score	Total No. of Patients	Duration (No. Cycles)	Number of Cycles Survival Advantage
Altman et al.	2019	14	2440	6	OS: 22 months ≥6 cycles vs. 17 months <6 cycles
Ivey et al.	2022	14	427	5	OS: HR = 0.81 CI: 0.59–1.08 for >5 cycles vs. ≤5 cycles
Weinrich et al.	2018	14	143	6	OS: HR = 0.443 CI: 0.270–0.727 for completed 6 cycles vs. no cycles
Kondo et al.	2022	14	290	15	OS: HR = 0.53 CI: 0.37–0.77 for ≥15 cycles vs. 10–14.5 cycles
Valle et al.	2014	22	985	6	OS: HR = 0.516 CI: 0.443–0.601 for ≥6 cycles vs. <6 cycles
Epelboym et al.	2017	14	522	6	OS: HR = 0.32 CI: 0.23–0.45 for ≥6 cycles vs. 0 cycles
Nitipir et al.	2021	14	42	6	OS: 8 months <6 cycles vs. 17.3 months >6 cycles (*p* = 0.6)
Chikhladze et al.	2019	14	251	6	OS: 27.7 months completed 6 cycles vs. 26.3 months 4–5 cycles vs. 14 months 1–3 cycles
S. H. Lee et al.	2023	14	218	6	DFS: HR = 1.17 CI: 0.79–1.72 for ≥6 cycles vs. <6 cycles
Habib et al.	2023	14	581	6	OS: HR = 0.69 CI: 0.54–0.87 ≥6 cycles vs. <6 cycles
Y. Murakami et al.	2013	14	104	10	OS: HR = 1.76 CI: 0.84–3.56 ≥10 cycles vs. <10 cycles
Woo Kim et al.	2017	14	113	6	OS: HR = 4.040 CI: 2.375–6.873 <6 cycles vs. ≥6 cycles
T. Conroy et al.	2022	22	493	≥12 mFOLFIRINOX or ≥6 GEM	OS: HR = 0.64 CI: 0.49–0.84 ≥12 cycles mFOLFIRINOX or ≥6 cycles GEM

**Table 2:** Combined time for all studies reporting on the effect of adjuvant therapy timing, relative dosage intensity, and specific type of chemotherapy regimen.

Article	Time to Adjuvant (Weeks)	Timing Survival Advantage	Relative Dosage Intensity (RDI)	In Reference to	Chemotherapy Regimen	Regimen Survival Advantage
Altman et al.	<12	-	-	-	GEM or FU	-
Chikhladze et al.	Median 8.5	-	-		GEM	-
Ivey et al.	-	-	-	-	FOLFIRINOX or GEM-based	OS: HR = 0.9 CI: 0.66–1.22 for FOLFIRINOX vs. GEM-based
Labori et al.	<8	-	-	-	FU and LV	-
S. H. Lee et al.	-	-	-	-	FOLFIRINOX	-
Perri et al.	-	-	-	-	FOLFIRINOX	-
Weinrich et al.	<6	-	-	-	GEM	-
S. Ei et al.	-	-	-	-	mFOLFIRINOX or GEM-based or 5-FU+LV	DFS: HR = 0.58 CI: 0.46–0.73 for mFOLFIRINOX vs. GEM DFS: HR = 0.88 CI: 0.73–1.06 for GEM+nab-PTX vs. GEM OS: HR = 0.82 CI: 0.68–0.98 for GEM-Cap vs. GEM OS: HR = 0.94 CI: 0.81–1.08 for GEM vs. 5-FU+LV
Habib et al.	-	-	-	-	GEM-based vs. GEM+5-FU	OS: HR = 0.77 CI: 0.56–1.07 for GEM+5-FU vs. GEM-based
Kondo et al.	2–6	-	-	-	FOLFIRINOX or GEM/nab-paclitaxel/S-1	-
Matsushima et al.	<7.3	OS: HR = 2.87 CI: 1.22–6.74 for AT ≥ 51 days	>72.3%	OS	S-1	-
Nitipir et al.	-	-	-	-	FOLFIRINOX	-
Valle et al.	≤12	OS: HR = 0.985 CI: 0.956–1.015 for AT ≤ 12 weeks	-	-	GEM or FU	-
Yabusaki et al.	-	-	≥80%	OS	S-1 vs. GEM	OS: S-1 95.0 months vs. GEM 26 months
PDQ Adult Treatment Editorial Board	-	-	-	-	FOLFIRINOX or GEM + CAP	-
Epelboym et al.	-	-	-	-	Gem-based or 5FU	OS: HR = 1.19 CI: 0.76–1.87 for GEM-based vs. 5FU
Bakens et al.	Median 6.7	-	-	-	-	-
A. T. Le et al.	Majority < 8	-	-	-	-	-
Sweigert et al.	<12	-	-	-	-	-
Turner et al.	<24	OS: HR = 0.744 CI: 0.699–0.792 for 6–12 weeks vs. no AT OS: HR = 0.736 CI: 0.671–0.808 for 12–24 weeks vs. no AT	-	-	-	-
Tzeng et al.	<8	-	-	-	GEM + Cisplatin	-
W. Wu et al.	Median 8.6	-	-	-	GEM + paclitaxel or 5FU based	-
N. Fu et al.	>4.9	OS: HR = 0.649 CI: 0.446–0.947 for AT > 34 days vs. <34 days	-	-	GEMCap vs. GEM	OS: HR = 0.547 CI: 0.332–0.904 for GEMCap vs. GEM
Sugumar et al.	≥6	OS: HR = 0.96 CI: 0.86–1.06 for 6–8 weeks AT OS: HR = 1.86 CI: 1.25–2.78 for 3–5 weeks	-	-	GEM or 5FU or FOLFIRINOX or CAP or S-1	-
Petrelli et al.	>6–8	OS: HR = 1 CI: 1–1.01 for AT 6–8 weeks vs. after 6–8 weeks	-	-	FOLFIRINOX or GEM	-
Y. Murakami et al.	≤2.9	OS: HR = 1.93 CI: 1.04–3.67 ACT ≤ 2.9 weeks vs. >2.9 weeks	-	-	GEM+S-1	-
Woo Kim et al.	>5	OS: HR = 1.880 CI: 1.107–3.191 ACT > 5 weeks vs. ≤5 weeks	-	-	GEM or 5-FU+LV or CAP based	-
Wan Kim et al.	>8	OS: HR = 1.63 CI: 1.13–2.35 ACT ≥ 8 weeks vs. <8 weeks	-	-	fluoropyrimidine-based or oxaliplatin-based	-
H. Saeed et al.	<8	OS: HR = 1.00 CI: 0.8–1.3 ACT < 8 weeks vs. 8–16 weeks	-	-	-	-
B. T. Xia et al.	>12	OS: HR = 0.96 CI: 0.51–1.80 ACT > 12 weeks vs. ≤12 weeks	-	-	GEM or GEM-CAP or FU	-
K. A. Mirkin et al.	>12	OS: HR = 1.00 CI: 0.90–1.12 ACT > 12 weeks vs. ≤12 weeks	-	-	-	-
J. P. Neoptolemos et al.	≤12	-	-	-	GEM or GEM-CAP	OS: HR = 0.82 CI: 0.68–0.98 GEM-CAP vs. GEM alone
M. A. Tempero et al.	≤12	-	-	-	GEM+nab-PTX or GEM	OS: HR = 0.82 CI: 0.68–0.99 GEM+nab-PTX vs. GEM alone
T. Conroy et al.	-	-	≥80%	OS: HR = 1.01 CI: 0.80–1.28 RDI ≥ 80% vs. <80%	mFOLFIRINOX or GEM	OS: HR = 0.73 CI: 0.58–0.92 mFOLFIRINOX vs. GEM alone

Note not all manuscripts presented in this table were used in the meta-analysis based on lack of HR or OR for data sets evaluated.

## Data Availability

All data is available on request to the corresponding authors.

## Supplementary Materials

The following supporting information can be downloaded at: https://www.mdpi.com/article/10.3390/cancers18121912/s1, Table S1. Methodological Quality Assessment of Included Studies Using the Methodological Index for Non-Randomized Studies (MINORS).

## Abbreviations

OS	overall survival
TTA	initiation of adjuvant chemotherapy
RDI	relative dosage intensity percentage
ACT	adjuvant chemotherapy
NAC	neo-adjuvant chemotherapy
PDAC	pancreatic ductal adenocarcinoma
CRT	chemoradiotherapy
ART	adjuvant radiotherapy
DFS	disease-free survival
MINORS	methodological index for non-randomized studies
HR	hazard ratios
