# Supplementary material for: Chemotherapy Completion as a Quality Metric in Resected Pancreatic Ductal Adenocarcinoma
Source: Cancers (Basel). 2026 Jun 11;18(12):1912. doi: 10.3390/cancers18121912 (PMC13296974; doi:10.3390/cancers18121912)
Supplement: Supplementary file 1 [file cancers-18-01912-s001.zip › cancers-4371109-supplementary.pdf]

Supplementary Table S1. Methodological Quality Assessment of Included Studies Using the Methodological Index for Non-Randomized Studies (MINORS)

Study	Year	Study Design	MINORS Score	Quality Category
Altman et al.	2019	Retrospective Cohort	14	Moderate
Ivey et al.	2022	Retrospective Cohort	14	Moderate
Labori et al.	2016	Population-Based Cohort	14	Moderate
Weinrich et al.	2018	Cohort Study	14	Moderate
Kondo et al.	2022	Retrospective Comparative	14	Moderate
Valle et al. (ESPAC-3)	2014	Randomized Trial Analysis	22	High
Epelboym et al.	2017	Retrospective Cohort	14	Moderate
Nitipir et al.	2021	Cohort Study	14	Moderate
Chikhladze et al.	2019	Retrospective Cohort	14	Moderate
Lee et al.	2023	Retrospective Cohort	14	Moderate
Habib et al.	2023	Retrospective Cohort	14	Moderate
Murakami et al.	2013	Retrospective Cohort	14	Moderate
Kim et al.	2017	Retrospective Cohort	14	Moderate
Conroy et al. (PRODIGE-24)	2022	Randomized Trial Follow-up	22	High
Matsushima et al.	2022	Retrospective Cohort	14	Moderate
Yabusaki et al.	2016	Retrospective Cohort	14	Moderate
Ei et al.	2023	Review/Comparative Analysis	14	Moderate

**Study**	**Year**	**Study Design**	**MINORS Score**	**Quality Category**
Perri et al.	2020	Retrospective Cohort	14	Moderate
Fu et al.	2022	Retrospective Cohort	14	Moderate
Bakens et al.	2016	Population-Based Study	14	Moderate
Tzeng et al.	2014	Retrospective Cohort	14	Moderate
Turner et al.	2020	Retrospective Cohort	14	Moderate
Wu et al.	2014	Retrospective Cohort	14	Moderate
Le et al.	2017	Retrospective Cohort	14	Moderate
Sweigert et al.	2020	Retrospective Cohort	14	Moderate
Xia et al.	2017	Multi-Institutional Cohort	14	Moderate
Mirkin et al.	2016	Retrospective Cohort	14	Moderate
Saeed et al.	2016	Registry Analysis	14	Moderate
Neoptolemos et al. (ESPAC-4)	2017	Randomized Phase III Trial	22	High
Tempero et al.	2023	Randomized Phase III Trial	22	High

Quality Categories:

• High Quality: MINORS ≥20

• Moderate Quality: MINORS 14–19

• Low Quality: MINORS <14

Overall Quality Summary:
Among the included studies, 4 (13.3%) were classified as high quality and 26 (86.7%) as moderate quality. No studies were classified as low quality. The overall body of evidence was therefore considered moderate-to-high methodological quality, although most studies were retrospective and susceptible to residual confounding and selection bias.
